# Angiotensin-Converting Enzyme Inhibitors and Angiotensin Receptor Blockers Withdrawal Is Associated with Higher Mortality in Hospitalized Patients with COVID-19

**DOI:** 10.3390/jcm10122642

**Published:** 2021-06-15

**Authors:** Emilia Roy-Vallejo, Aquilino Sánchez Purificación, José David Torres Peña, Beatriz Sánchez Moreno, Francisco Arnalich, María José García Blanco, José López Miranda, Juan Luis Romero-Cabrera, Carmen Rosario Herrero Gil, José Bascunana, Manuel Rubio-Rivas, Sara Pintos Otero, Verónica Martínez Sempere, Jesús Ballano Rodríguez-Solís, Ricardo Gil Sánchez, Jairo Luque del Pino, Amara González Noya, María Sierra Navas-Alcántara, Begoña Cortés Rodríguez, José Nicolás Alcalá, Ana Suárez-Lombraña, Jorge Andrés Soler, Ricardo Gómez-Huelgas, José Manuel Casas-Rojo, Jesús Millán Núñez-Cortés, on behalf of the SEMI-COVID-19 Network

**Affiliations:** 1 Department of Internal Medicine, La Princesa University Hospital, Healthcare Research Institute-La Princesa Biomedical Research Foundation, 28006 Madrid, Spain; eroyvallejo@gmail.com (E.R.-V.); mariajgarciablanco@gmail.com (M.J.G.B.); 2 Department of Internal Medicine, La Paz University Hospital, Hospital La Paz Institute for Health Research (IdiPAZ), Autonomous University of Madrid, 28046 Madrid, Spain; aquilino.sanchez@salud.madrid.org (A.S.P.); farnalich@salud.madrid.org (F.A.); carmen_ela92@hotmail.com (C.R.H.G.); 3 Lipids and Atherosclerosis Unit, Department of Internal Medicine, Maimonides Biomedical Research Institute of Cordoba (IMIBIC), Reina Sofia University Hospital, University of Córdoba, 14004 Córdoba, Spain; jlopezmir@uco.es (J.L.M.); juanluroca855@gmail.com (J.L.R.-C.); 4 CIBER Physiopathology of Obesity and Nutrition (CIBEROBN), Carlos III Institute of Health, 28029 Madrid, Spain; 5 Internal Medicine Department, 12 de Octubre University Hospital, 28041 Madrid, Spain; josebascumor@gmail.com; 6 Internal Medicine Department, Bellvitge University Hospital-IDIBELL, L’Hospitalet de Llobregat, 08907 Barcelona, Spain; mrubio@bellvitgehospital.cat; 7 Internal Medicine Department, Zamora Hospital Complex, 49022 Zamora, Spain; sarapintosotero@gmail.com; 8 Internal Medicine Department, San Juan de Alicante University Hospital, 03550 San Juan de Alicante, Spain; veronicamartinez_18@hotmail.com; 9 Internal Medicine Department, Henares Hospital, 28822 Coslada, Spain; jesus.ballano@salud.madrid.org; 10 Internal Medicine Department, La Fe University Hospital, 46026 Valencia, Spain; rigilsan@gmail.com; 11 Internal Medicine Department, Costa del Sol Hospital, 29603 Marbella, Spain; jairo_malaga@hotmail.com; 12 Internal Medicine Department, Ourense University Hospital Complex, 32005 Ourense, Spain; amara.gonzalez.noya@sergas.es; 13 Internal Medicine Department, Infanta Margarita Hospital, 14940 Cabra, Spain; maria.sierra.navas@gmail.com; 14 Internal Medicine Department, Alto Guadalquivir Hospital, 23740 Andújar, Spain; begocortesrod@gmail.com; 15 Internal Medicine Department, Pozoblanco Hospital, 14400 Pozoblanco, Spain; jnalcala58@hotmail.com; 16 Internal Medicine Department, Platón Hospital, 08006 Barcelona, Spain; anasuarezlombrana@gmail.com; 17 Internal Medicine Department, Castellón University General Hospital, 12004 Castellón de la Plana, Spain; jandressoler@hotmail.com; 18 Internal Medicine Department, Regional University Hospital of Málaga, Biomedical Research Institute of Málaga (IBIMA), University of Málaga (UMA), 29010 Málaga, Spain; ricardogomezhuelgas@hotmail.com; 19 Internal Medicine Department, Infanta Cristina University Hospital, 28981 Parla, Spain; jm.casas@gmail.com; 20 Internal Medicine Department, Gregorio Marañón University Hospital, 28007 Madrid, Spain; jesus.millan@salud.madrid.org

**Keywords:** COVID-19, ACEI, ARB, prognosis, MACE

## Abstract

Our main aim was to describe the effect on the severity of ACEI (angiotensin-converting enzyme inhibitor) and ARB (angiotensin II receptor blocker) during COVID-19 hospitalization. A retrospective, observational, multicenter study evaluating hospitalized patients with COVID-19 treated with ACEI/ARB. The primary endpoint was the incidence of the composite outcome of prognosis (IMV (invasive mechanical ventilation), NIMV (non-invasive mechanical ventilation), ICU admission (intensive care unit), and/or all-cause mortality). We evaluated both outcomes in patients whose treatment with ACEI/ARB was continued or withdrawn. Between February and June 2020, 11,205 patients were included, mean age 67 years (SD = 16.3) and 43.1% female; 2162 patients received ACEI/ARB treatment. ACEI/ARB treatment showed lower all-cause mortality (*p* < 0.0001). Hypertensive patients in the ACEI/ARB group had better results in IMV, ICU admission, and the composite outcome of prognosis (*p* < 0.0001 for all). No differences were found in the incidence of major adverse cardiovascular events. Patients previously treated with ACEI/ARB continuing treatment during hospitalization had a lower incidence of the composite outcome of prognosis than those whose treatment was withdrawn (RR 0.67, 95%CI 0.63–0.76). ARB was associated with better survival than ACEI (HR 0.77, 95%CI 0.62–0.96). ACEI/ARB treatment during COVID-19 hospitalization was associated with protection on mortality. The benefits were greater in hypertensive, those who continued treatment, and those taking ARB.

## 1. Introduction

It has been suggested that the renin-angiotensin-aldosterone system (RAAS) has a relevant role in the pathogenesis of COVID-19 caused by SARS-CoV-2. The virus can infect host cells through interaction with angiotensin-converting enzyme 2 in the respiratory epithelium [1]. Therefore, there has been concern about risk related to the use of RAAS blockers in the context of COVID-19.

This concern is even more relevant given that a large part of patients with COVID-19 is hypertensive. Hypertension and cardiovascular disease (pathologies in which these drugs are usually prescribed) are more common in COVID-19 patients with severe disease [2]. Several publications have provided data on the effect of angiotensin-converting enzyme inhibitors (ACEI) or angiotensin receptor blockers (ARB) in the context of SARS-CoV-2 infection. None of them have shown a negative effect on COVID-19 mortality and incidence, and some described an improvement in survival [1,3,4,5]. However, the effect of maintaining or discontinuing ACEI/ARB during hospitalization is still uncertain. To date, the main scientific societies advise against the interruption of these drugs due to the negative consequences that may arise from their suspension in patients with cardiovascular diseases [6,7].

SARS-CoV-2 has high morbidity and mortality: as of 10 March 2021, nearly 118 million (117,764,619) cases of COVID-19 had been diagnosed, and over 2,613,747 people had died [8]. Therefore, the analysis of large datasets is essential to establish patient profiles so that the currently available therapeutic tools can be used and it can be ascertained whether treatment can alter the course of the disease.

The main objective of this work was to evaluate how treatment with ACEI/ARB in hospitalized patients with COVID-19 modifies mortality and respiratory complications. As secondary objectives, we analyzed whether treatment with these medications during hospitalization had an effect on the incidence of major adverse cardiovascular events (MACE) and the effect of withdrawing these drugs during hospitalization.

## 2. Materials and Methods

### 2.1. Study Design

This is an observational, retrospective, multicenter cohort study with the participation of 150 Spanish hospitals that is part of the SEMI-COVID-19 Network, an open initiative of the Spanish Society of Internal Medicine (SEMI). The registry enrolls consecutive patients with a positive SARS-CoV-2 real-time polymerase chain reaction (RT-PCR) of a nasopharyngeal swab admitted to participating hospitals in Spain from February 2020 until 4 June 2020.

**Figure 1:**
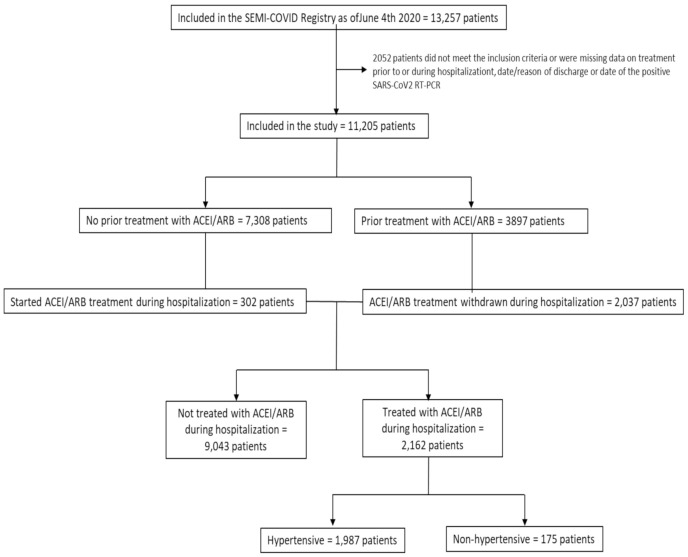
Patient inclusion flowchart. RT-PCR: real-time polymerase chain reaction, ACEI: angiotensin-converting enzyme inhibitor, ARB: angiotensin II receptor blocker.

The inclusion criteria for the study were: age > 18 years, positive RT-PCR for SARS-CoV-2, admission to any participating hospital, and availability of a minimum set of demographic data (age, sex, race, and onset of symptoms). We excluded patients who remained hospitalized as of 4 June 2020, those who did not have data available on treatment with ACEI/ARB before and during hospitalization, or the date of the first positive RT-PCR recorded (Figure 1).

Patients were classified in the ACEI/ARB group if they received at least one dose of any ACEI/ARB during hospitalization. The total dose of ACEI/ARB was not recorded in the registry. Previous ACEI/ARB use was determined based on the last entry in the medical chart.

Each patient’s management and treatment was the responsibility of the attending physicians based on their hospital protocols, the recommendations of the Spanish Agency of Medicines and Medical Products, and their clinical judgment.

### 2.2. Data Collection

All data were collected from the medical charts and included in the registry’s encrypted online database. An independent external agency monitored and reviewed the information for inconsistencies. Full details on the SEMI-COVID-19 Registry have been described previously [9].

### 2.3. Main Outcomes

In order to evaluate the primary objective, we created a composite outcome of prognosis that included the need for invasive mechanical ventilation (IMV), non-invasive mechanical ventilation (NIMV), intensive care unit (ICU) admission, and/or all-cause mortality.

As a secondary objective, we assessed the effect of ACEI/ARB treatment on cardiovascular risk in COVID-19 patients by establishing the composite outcome of major adverse cardiovascular events (MACE), which included incidence of myocardial infarction (MI), heart failure (HF), stroke, and/or any arrhythmia (atrial or ventricular). Cardiovascular mortality was not included because this data was not available. We also evaluated the composite outcome of prognosis and MACE in those that continued or discontinued ACEI/ARB treatment during hospitalization.

### 2.4. Statistical Analysis

Quantitative variables were expressed as mean and standard deviation (SD) or median and interquartile range (IQR) if they did not follow a Gaussian distribution. Categorical variables were expressed as frequencies and percentages. Baseline characteristics were compared between the ACEI/ARB and non-ACEI/ARB groups using Student’s *t*-test or the Wilcoxon test for continuous variables or the chi-square test for qualitative variables.

We compared the incidence of both the aforementioned composite outcomes and of each individual event during hospitalization between the ACEI/ARB and non-ACEI/ARB groups using logistic regression models and estimating adjusted relative risks using the marginal standardization method. On each regression analysis, several predictors were considered to be possible modifying and/or confounding factors: age, sex, race, smoking, alcohol use, hypertension, dyslipidemia, diabetes mellitus, obesity, chronic kidney disease, chronic heart failure, prior treatment with ACEI/ARB, Charlson Comorbidity Index, and in-hospital treatment with tocilizumab or corticosteroids. Modifiers were first selected by statistical criteria (*p* < 0.05) using likelihood-ratio tests, and then confounders were chosen by comparing all possible subsets of the maximum model, which included all the significant modifiers found in the first place. For the composite outcomes, we selected the most parsimonious models that did not result in a clinically significant change (<5%) on the odds ratio in comparison to the maximal reference model. We could apply this restrictive threshold as there were many models to compare, given the number of predictors evaluated.

Early in the COVID-19 pandemic, a significant number of patients had treatment withdrawn during hospitalization. Using logistic regression models, we analyzed whether the effect of ACEI/ARB use was sustained in patients whose treatment was withdrawn compared to those whose ACEI/ARB treatment was continued. On this analysis, we considered the same composite outcomes of prognosis and MACE.

Lastly, we performed a survival analysis to evaluate the effect of ACEI/ARB use and of continuing/withdrawing this treatment during hospitalization, comparing Kaplan–Meier curves with the log-rank test. In addition, Cox proportional-hazards models allowed for including additional covariates in a similar manner to the logistic regression analysis described above. These results were expressed as hazard ratios after confirming that the proportional hazard assumption was met.

All statistical analyses were performed using Stata software (version 15.0, Stata Corp, College Station, TX, USA). A two-tailed *p*-value of 5% was established as the threshold of statistical significance.

### 2.5. Ethics

The SEMI-COVID-19 Registry was approved by the Provincial Research Ethics Committee of Málaga (code: “Registro SEMI-COVID-19”, approved 27 March 2020). Given the state of emergency declared during the pandemic, it was only mandatory for patients to provide verbal consent. This manuscript was written following the recommendations of the Strengthening the Reporting of Observational studies in Epidemiology (STROBE) statement (STROBE Checklist in Supplementary Materials).

## 3. Results

### 3.1. Study Population

A total of 11,205 patients were included in the study (Figure 1). The mean age of patients was 67 years (SD = 16.3), 43.1% were female, and 89.3% were Caucasian. During hospitalization, 2162 (19.3%) participants were treated with ACEI/ARB. Subjects in the ACEI/ARB group were older (72.5 vs. 65.7 years, *p* < 0.0001), more frequently male (59.1% vs. 56.3%, *p* = 0.018), Caucasian (93.8% vs. 88.2%, *p* < 0.001), more likely active smokers (15.4% vs. 5.4%, *p* < 0.001) and with an alcohol use disorder (6.1% vs. 4.4%, *p* = 0.001) than those in the non-ACEI/ARB group. Furthermore, they had more comorbidities (age-adjusted Charlson Comorbidity Index of 4.4 points (SD = 2.5) vs. 3.4 points (SD = 2.7), *p* < 0.001), with hypertension being the most prevalent (92.1% vs. 39.7%, *p* < 0.001). Prior to hospitalization, 41.5% of patients were treated with ACEI and 47.6% with ARB in the ACEI/ARB group. In the non-ACEI/ARB group, 16.9% of patients were treated with ACEI and 19% with ARB. The main reason for ACEI/ARB treatment (92% of the patients) was hypertension, while 8% were taking these drugs for other diseases. Baseline characteristics are shown in Table 1 (treatment and analytical data in Supplementary Table S1).

### 3.2. Outcomes of Prognosis

At least one of the events of the composite outcome of prognosis (IMV, NIMV, ICU admission, or death) occurred in 569 patients (27.0%) in the ACEI/ARB group and in 2443 (27.6%) in the non-ACEI/ARB group (*p* = 0.6). The results of the univariate and multivariate analyses are shown in Table 2.

In the study population as a whole, the multivariate analysis revealed that patients treated with ACEI/ARB during hospitalization had lower mortality risk (RR 0.65, 95%CI 0.59–0.72) with no significant effect on the probability of needing NIMV (RR 1.13, 95%CI 0.9–1.43).

We found different effects in hypertensive and non-hypertensive patients on the remaining components of the composite outcome of prognosis. In the hypertensive subgroup, treatment with ACEI/ARB during hospitalization was associated with a lower prevalence of the composite variable of prognosis (RR 0.68, 95%CI 0.62–0.75), IMV (RR 0.50, 95%CI 0.39–0.64), and ICU admission (RR 0.57, 95%CI 0.46–0.71). In the normotensive subgroup, it showed a neutral effect on the composite variable of prognosis (RR 1.12, 95%CI 0.90–1.38), and it was associated with a higher risk of IMV (RR 2.07, 95%CI 1.42–3.02) and ICU admission (RR 1.76, 95%CI 1.23–2.52). Neither NIMV nor all-cause mortality had a significant relationship with hypertension or any other predictors. Reduced regression model in Supplementary Table S2.

**Figure 2:**
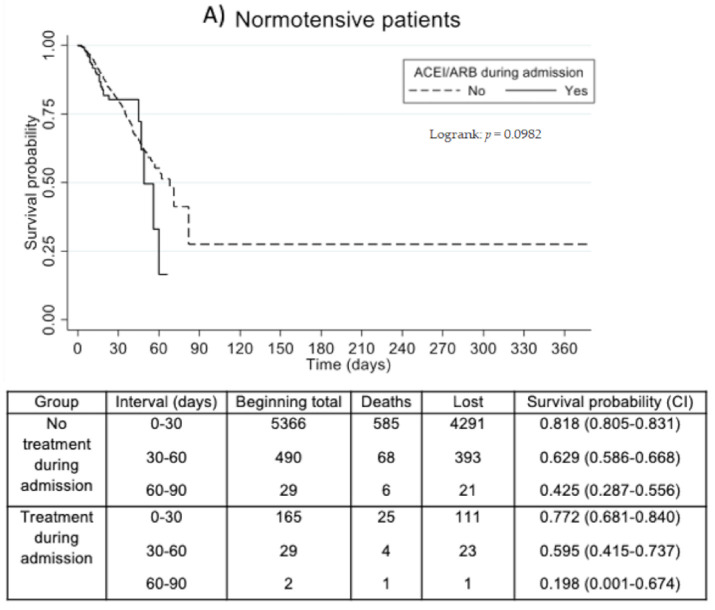

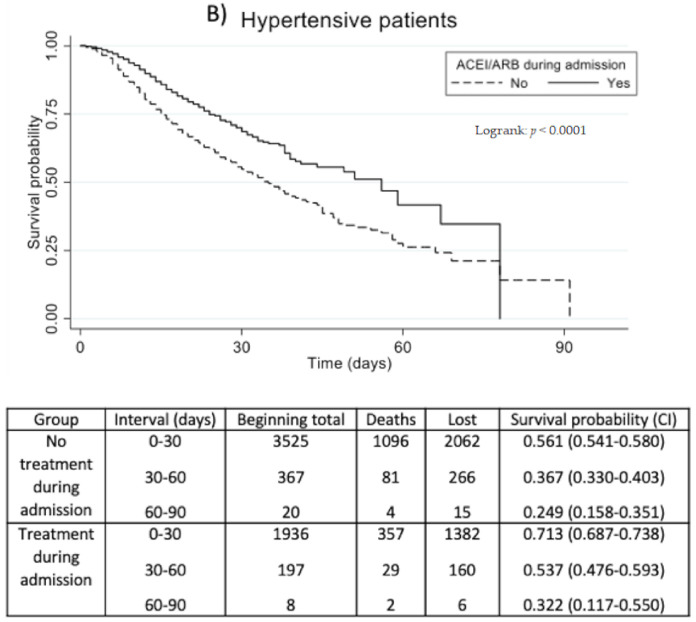
Effect on survival of treatment with ACEI/ARB during hospitalization. (**A**) Kaplan–Meier survival analysis for normotensive patients; (**B**) Kaplan–Meier survival analysis for hypertensive patients. Treatment with ACEI/ARB in solid lines; non-ACEI/ARB group in dotted lines. ACEI/ARBACEI: Angiotensin-Converting Enzyme Inhibitor. ARB: Angiotensin Receptor Blocker. Statistical significance was determined with a log-rank test.

On the survival analysis, significant differences between the ACEI/ARB and non-ACEI/ARB groups were found when patients were classified by hypertensive status (Figure 2).

We also performed a Cox regression, and since it did not meet the proportionality assumption, we calculated the HR for different periods of time, starting with the onset of symptoms. It showed a protective effect of ACEI/ARB treatment that progressively decreased over time (Table 3).

### 3.3. Major Adverse Cardiovascular Events

In the ACEI/ARB group, 12% of patients had a MACE. This percentage was significantly higher than the 9.2% found in the non-ACEI/ARB group (*p* = 0.001). A description of the major adverse cardiovascular events by group is shown in Table 4.

After adjusting, treatment with ACEI/ARB during hospitalization was found to be neither protective nor harmful regarding MACE (RR 0.94, 95%CI 0.81–1.09) or any of its components (multivariate analysis in Table 4). Reduced regression model in Supplementary Table S2.

### 3.4. ACEI/ARB Continuation Versus Withdrawal during Hospitalization

A total of 3897 patients were receiving ACEI and/or ARB prior to hospitalization. Of them, 1860 continued treatment. The ACEI/ARB group had a lower probability of the composite variable of prognosis (RR 0.66, 95%CI 0.6–0.72) and MACE (RR 0.75, 95%CI 0.64–0.88) compared to the non-ACEI/ARB group. This effect was sustained on the multivariate analysis for the composite variable of prognosis (RR 0.67, 95%CI 0.63–0.76) but not for MACE (RR 0.86, 95%CI 0.73–1.01).

**Figure 3:**
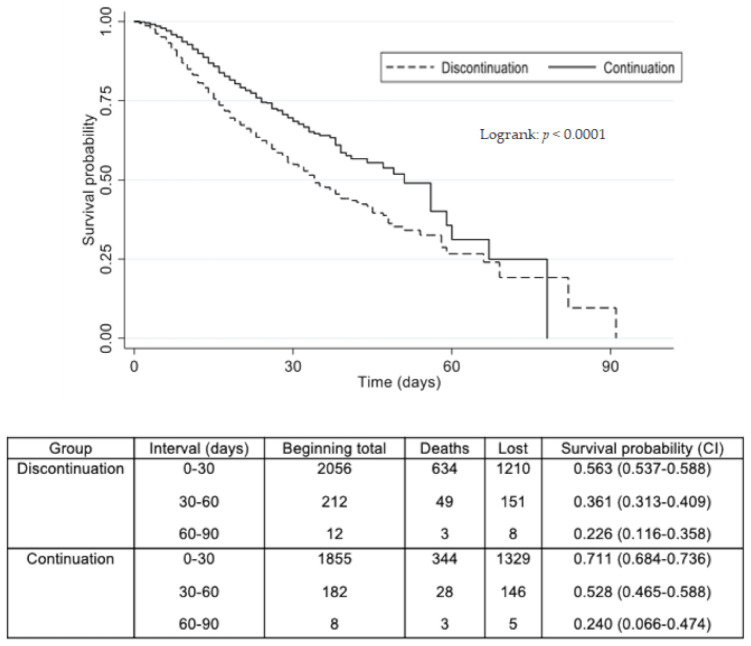
Effect on survival of discontinuation vs. continuation of ACEI/ARB treatment during hospitalization. Kaplan–Meier survival analysis comparing continuation of ACEI/ARB (solid lines) with its withdrawing (dotted lines). ACEI: angiotensin-converting enzyme inhibitor. ARB: angiotensin receptor blocker. Statistical significance was determined with a log-rank test.

There was also a significant difference in the survival analysis between the ACEI/ARB and non-ACEI/ARB groups (Figure 3). Continuing ACEI/ARB treatment led to a lower risk of death on the Cox regression, but this protective effect also diminished over time (Table 3).

### 3.5. Comparison between ACEI and ARB

When comparing the effects of ACEI versus ARB, no significant differences were found on the univariate or multivariate analyses in terms of the composite variable of prognosis or MACE. Nevertheless, a more favorable effect on survival was found with ARB after adjusting for confounding factors (Table 3).

## 4. Discussion

In this series, we analyzed the effects of ACEI and ARB in a large number of patients admitted to Spanish hospitals for COVID-19 between February and June 2020. The most relevant finding of this study is that ACEI/ARB treatment during hospitalization was associated with a 30% reduction in mortality.

It is especially relevant to highlight the benefits of ACEI/ARB in hypertensive patients with COVID-19. Indeed, 92.1% of those who received these drugs during hospitalization were hypertensive, and results showed a relative risk reduction (RRR) of 32% on the composite variable of prognosis in these patients. There was also a significant benefit observed on other variables: 50% RRR for IMV, 43% for ICU admission, and 35% for all-cause mortality.

The results obtained with ACEI/ARB are noteworthy if we consider that the group of patients who received these drugs had higher mean age, greater comorbidity, and higher prevalence of cardiovascular diseases and risk factors). It has been published in this registry [10], and in other series [11] that age, hypertension, and previous cardiovascular disease are factors associated with a worse prognosis and higher risk of mortality in patients with SARS-CoV-2 infections.

Our results are consistent with other published series of COVID-19 patients previously treated with ACEI/ARB. Data reported by Reynolds et al. [1] and Mancia et al. [5] showed no association between ARB/ACEI use and risk of infection for COVID-19 or a severe course of the disease. In both studies, the treated group also had a worse clinical profile and higher prevalence of cardiovascular diseases. Other large series also showed this neutral effect of ACEI/ARB in SARS-CoV-2 infection [3,4]. In addition, no difference in the incidence or severity of COVID-19 has been demonstrated in series comparing ACEI/ARB with other antihypertensives [1,12,13]. Previous meta-analyses have confirmed the absence of a harmful effect of these drugs [14,15,16].

To date, there is insufficient evidence of the effect of ACEI/ARB used during COVID-19 hospitalization. A randomized clinical trial found no difference in mortality between those who were maintained on ACEI/ARB and those who were discontinued [17]. However, two small retrospective series described lower mortality in patients that continued ACEI/ARB during hospitalization [18,19].

As in our survival analysis, previous studies analyzing 28-days mortality described the benefit of maintaining ACEI/ARB during COVID-19 hospitalization [20,21]. These results suggest that ACEI/ARB withdrawal can lead to a greater risk for complications and mortality for most patients. Some authors have suggested that the benefit of ACEI/ARB treatment is based on the lower inflammatory response during acute lung injury due to blockage of the RAAS, especially with ARB [18,22].

Our study also includes a small proportion of patients (8%; 170 patients) who received ACEI/ARB for diseases other than hypertension, including heart failure, coronary heart disease, and chronic kidney disease. However, we were not able to determine the cause of treatment in a significant number of patients. In many of them, ACEI/ARB might cause adverse effects during hospitalization, such as hypotension or acute renal failure. This could explain the worse prognosis of this subgroup and why the results observed in the entire study population are not as unequivocal as in the hypertensive population. Acute kidney injury has been reported in a small series of hospitalized patients with COVID-19 in whom treatment with ACEI/ARB was maintained. [23] For this reason, some authors exclude patients with severe organ dysfunction and patients with hypotension from their series [20,21].

No benefits were observed in the reduction in MACE. In the published clinical trial, Lopes et al. [17] also found no difference on the MACE analyzed. The beneficial effects of ACEI/ARB have been widely demonstrated for each of the cardiovascular events included in MACE [24]. The higher mean age and prevalence of cardiovascular risk factors and cardiovascular diseases in the ACEI/ARB group may explain the greater incidence of cardiovascular events observed. This result suggests that the benefit achieved with ACEI/ARB during COVID-19 hospitalization is not cardiovascular but rather of another nature.

Though no differences were noted between ACEI and ARB on the main composite outcomes, a significant difference in survival was found in the group that received ARB (23% reduction in mortality). A recently published article found a similar result with ARB treatment [10]. Although current data is limited, inconclusive, and based on animal models, there is some evidence that ARB may have a beneficial effect in reducing angiotensin II-induced alveolar permeability, an effect not observed with ACEI [22].

Our study has several strengths. First, a large number of patients were recruited. In fact, it is one of the largest series of patients with COVID-19 published to date. As a nationwide study, it quite accurately reflects the reality in Spain during the first months of the pandemic and avoids the biases of other published series that include data from only a few centers. Second, we included hypertensive patients as well as all who received ACEI/ARB during hospitalization for COVID-19. Unlike other series and in addition to mortality, major respiratory and cardiovascular complications were included as one of the main objectives of this study, and we analyzed the effect that discontinuation of these drugs had on these complications.

This study also has several limitations. First, it is retrospective and observational. Second, the decision to maintain/withdraw treatment depended on each hospital’s protocol or each attending physician’s judgment. This could lead to selection bias not only in favor of ACEI/ARB, withdrawing these drugs in patients with worse prognosis, but also against them, maintaining them in non-hypertensive patients with complications that can be worsened by ACEI/ARB. The reason for discontinuing ACEI/ARB was not available in the SEMI-COVID-19 Registry. However, early in the pandemic, the uncertainty about the safety of ACEI/ARB during SARS-CoV-2 infection could have led to their withdrawal in many cases. Third, we do not know which ACEI/ARB drugs were used, at what doses, and for how long. Lastly, this series mainly comprises Caucasian patients, so our results may not be extrapolated to other populations.

## 5. Conclusions

In conclusion, our results suggest that ACEI/ARB should not be routinely withdrawn in patients hospitalized for COVID-19, especially in hypertensive patients. However, to confirm these results, more prospective and randomized controlled trials are needed. This work also points to an exciting field of research to be explored further: analysis of the molecular mechanisms that underlie the possible protective effect of ACEI/ARB against SARS-CoV-2.

## Figures and Tables

**Table 1:** Demographic and clinical data.

	Total Population (*n* = 11,205)	Non-ACEI/ARB Group (*n* = 9043)	ACEI/ARB Group (*n* = 2162)	*p*-Value
Age (years): mean (SD)	67.0 (16.3)	65.7 (16.8)	72.5 (12.5)	<0.0001
Female sex (%)	4827/11,190 (43.1%)	3944/9030 (43.7%)	883/2160 (40.9%)	0.018
Race/Ethnicity (%)				
Caucasian	9831/11,014 (89.3%)	7836/8886 (88.2%)	1995/2128 (93.8%)	<0.001 *
African	45/11,014 (0.4%)	36/8886 (0.4%)	9/2128 (0.4%)	
Latin American	990/11,014 (9.0%)	892/8886 (10.0%)	98/2128 (4.6%)	
Asian	50/11,014 (0.5%)	43/8886 (0.5)	7/2128 (0.3%)	
Other	98/11,014 (0.9%)	79/8886 (0.9%)	19/2128 (0.9%)	
Smoking (%)				
Non-smoker	7438/10,699 (69.5%)	6125/8617 (71.1%)	1313/2082 (63.1%)	<0.001 *
Former smoker	2686/10,699 (25.0%)	2030/8617 (23.6%)	656/2082 (31.5%)	
Active smoker	575%10,699 (5.4%)	462/8617 (5.4%)	113/2082 (15.4%)	
Alcohol use disorder (%)	516/10,887 (4.7%)	387/8780 (4.4%)	129/2107 (6.1%)	0.001
Comorbidities (%)				
Hypertension	5576/11,190 (49.8%)	3589/9033 (39.7%)	1987/2157 (92.1%)	<0.001
Dyslipidemia	4415/11,189 (39.5%)	3231/9029 (35.8%)	1184/2160 (54.8%)	<0.001
Diabetes mellitus	2095/11,178 (18.7%)	1464/9022 (16.2%)	631/2156 (29.3%)	<0.001
Obesity	2186/10,212 (21.4%)	1618/8242 (19.6%)	568/1970 (28.8%)	<0.001
Heart failure	811/11,186 (7.3%)	594/9030 (6.6%)	217/2156 (10.1%)	<0.001
Ischemic heart disease	880/11,193 (7.9%)	591/9032 (6.5%)	289/2161 (13.4%)	<0.001
Cerebrovascular disease	797/11,173 (7.1%)	598/9018 (6.6%)	199/2155 (9.2%)	<0.001
Peripheral artery disease	523/11,183 (4.7%)	387/9026 (4.3%)	136/2157 (6.3%)	<0.001
Chronic kidney disease	665/11,183 (5.9%)	491/9026 (5.4%)	174/2157 (8.1%)	<0.001
Age-adjusted Charlson Comorbidity Index: points (SD)	3.6 (2.7)	3.4 (2.7)	4.4 (2.47)	<0.0001
Previous treatment (%)				
ACEI	1890/11,205 (16,9%)	993/9043 (11.0%)	897/2162 (41.5%)	<0.001
ARB	2133/11,205 (19.0%)	1103/9043 (12.2%)	1030/2162 (47.6%)	<0.001

SD: standard deviation, IQR: interquartile range, ACEI: angiotensin-converting enzyme inhibitor, ARB: angiotensin II receptor blocker. * The *p*-value refers to Caucasian and Latin American patients and all categories of smoking.

**Table 2:** Univariate and multivariate analysis of prognosis outcomes by treatment group.

Outcome	Non-ACEI/ARB Group	ACEI/ARB Group	Univariate		Multivariate	
			RR (95%CI)	*p*-Value	RR (95%CI)	*p*-Value
Composite variable of prognosis: IMV, NIMV, ICU admission, or death *	2443/8854 (27.6%)	569/2107 (27.0%)	0.98 (0.91–1.06)	0.6064	Normotensive: 1.12 (0.90–1.38) Hypertensive: 0.68 (0.62–0.75)	0.3085 <0.0001
IMV *	622/9024 (6.9%)	115/2157 (5.3%)	0.77 (0.64–0.94)	0.0100	Normotensive: 2.07 (1.42–3.02) Hypertensive: 0.50 (0.39–0.64)	0.0002 <0.0001
NIMV	396/9026 (4.4%)	130/2156 (6.0%)	1.37 (1.13–1.67)	0.0015	1.13 (0.90–1.43)	0.2908
ICU admission *	767/9035 (8.5%)	148/2161 (6.8%)	0.81 (0.68–0.96)	0.0140	Normotensive: 1.76 (1.23–2.52) Hypertensive: 0.57 (0.46–0.71)	0.0019 <0.0001
Death	1897/8853 (21.4%)	436/2105 (20.7%)	0.97 (0.88–1.06)	0.4897	0.65 (0.59–0.72)	<0.0001

* A significant relationship with hypertension was found on the multivariate analysis. RR: relative risk, 95%CI: 95% confidence interval, ACEI: angiotensin-converting enzyme inhibitor, ARB: angiotensin II receptor blocker, IMV: invasive mechanical ventilation, NIMV: non-invasive mechanical ventilation, ICU: intensive care unit.

**Table 3:** Survival analysis: hazard ratios (HR) estimated via Cox regression.

Comparison	Adjusted HR	95%CI	*p*-Value
ACEI/ARB vs. non-ACEI/ARB during hospitalization	From the onset of symptoms: At 7 days: 0.57 At 30 days: 0.68	0.49–0.66 0.55–0.85	<0.001 0.001
ACEI/ARB continued vs. withdrawn	From the onset of symptoms: At 7 days: 0.52 At 30 days: 0.79	0.44–0.60 0.60–1.05	<0.001 0.110
ARB vs. ACEI	0.77	0.62–0.96	0.027

HR: hazard ratio, 95%CI: 95% confidence interval, ACEI: angiotensin-converting enzyme inhibitor, ARB: angiotensin II receptor blocker.

**Table 4:** Univariate and multivariate analysis of MACE outcomes by treatment group.

Outcome	Non-ACEI/ARB Group	ACEI/ARB Group	Univariate		Multivariate	
			RR (95%CI)	*p*-Value	RR (95%CI)	*p*-Value
Major adverse cardiovascular events (MACE): MI, HF, stroke, arrhythmia	827/8986 (9.2%)	257/2144 (12.0%)	1.30 (1.14–1.49)	0.0001	0.94 (0.81–1.09)	0.4211
MI	60/9005 (0.7%)	28/2151 (1.3%)	1.95 (1.23–3.05)	0.0043	1.64 (0.93–2.89)	0.0877
HF	504/9010 (5.6%)	160/2150 (7.4%)	1.33 (1.12–1.58)	0.0014	1.03 (0.85–1.26)	0.7597
Stroke	51/9003 (0.6%)	18/2153 (0.8%)	1.48 (0.86–2.52)	0.2005	0.90 (0.46–1.73)	0.7435
Arrhythmia	347/9002 (3.9%)	104/2152 (4.8%)	1.25 (1.01–1.55)	0.0446	0.86 (0.66–1.11)	0.2335

RR: relative risk, 95%CI: 95% confidence interval, ACEI: angiotensin-converting enzyme inhibitor, ARB: angiotensin II receptor blocker, MI: myocardial infarction, HF: heart failure.

## Data Availability

All data will be available under reasonable request.

## Supplementary Materials

The following are available online at https://www.mdpi.com/article/10.3390/jcm10122642/s1, Table S1: Laboratory and treatment data according to ACEI/ARB treatment group, Table S2: Reduced logistic regression models, Supplementary Materials: list of the SEMI-COVID Network members; STROBE Checklist.
